# Dreams of Teeth Falling Out: An Empirical Investigation of Physiological and Psychological Correlates

**DOI:** 10.3389/fpsyg.2018.01812

**Published:** 2018-09-26

**Authors:** Naama Rozen, Nirit Soffer-Dudek

**Affiliations:** Consciousness and Psychopathology Laboratory, Department of Psychology, Ben-Gurion University of the Negev, Beersheba, Israel

**Keywords:** typical dreams, sleep bruxism, teeth grinding, continuity hypothesis, psychopathology

## Abstract

Teeth dreams (TD), i.e., dreams of teeth falling out or rotting, are one of the most common and universal typical dream themes, yet their source remains unknown and they have rarely been studied empirically. They are especially enigmatic as they do not readily fall under the rubric of the “continuity hypothesis”, i.e., dreams of current and salient waking-life experiences. The aim of the present study was to explore two possible hypotheses for the origin of TD; specifically, TD as incorporation of dental irritation into dreaming, and TD as a symbolic manifestation of psychological distress. Dream themes, dental irritation, psychological distress, and sleep quality were assessed among 210 undergraduates. TD were related to dental irritation (specifically, tension sensations in the teeth, gums, or jaws upon awakening), whereas other dream types were not. Conversely, TD were unrelated to psychological distress, whereas other dream types were (specifically, dreams of being smothered and dreams of falling). This disparity in the correlates of TD existed despite a small but significant relationship between psychological distress and dental irritation. Albeit preliminary, the present findings support the dental irritation hypothesis and do not support the symbolic hypothesis regarding the origins of TD. Research on TD portrays one path through which the mind may distort somatosensory stimuli and incorporate them into dreams as a vivid and emotionally salient image; these preliminary findings highlight the potential of studying TD in order to broaden our understanding of the cognitive mechanisms governing dream production.

## Introduction

Dreams of teeth falling out, losing one’s teeth, or teeth breaking or rotting, constitute one of the most prevalent typical dream themes. For example, in one study, 39.0% of respondents reported that they had experienced teeth dreams (TD) at least once, 16.2% reported that their TD were recurrent, and 8.2% reported that their TD were regular (Yu, 2012). TD are so prevalent that they have even received portrayals in popular media, such as the Walt Disney movie “Inside Out" (Rivera and Docter, 2015), in which they were depicted as a manifestation of distress (a reasonable hypothesis which we will discuss further below). The commonness of TD is somewhat inexplicable, as it is incompatible with the “continuity hypothesis", according to which, we dream of our waking concerns and waking life-experiences (e.g., Domhoff, 1996). In other words, it is difficult to explain why so many people dream, sometimes regularly, of the experience of teeth falling out, breaking, or rotting, experiences which are not particularly common in waking life for adults. Understanding this disparity may be important for understanding the mechanisms governing dream production. Nevertheless, this topic has hardly received empirical attention, even within the narrow field of dream research.

Because TD are so common and universal, there have been several attempts to provide interpretations for them. Perhaps the earliest documented interpretation was given in Ancient Greece by Artemidorus, who meticulously divided the oral cavity to several components (e.g., molars, incisors, right and left side of the mouth) and gave each part a specific meaning. For example, he related losing teeth in a dream to the payment of debts (Coolidge, 2006). Soon after, in the Jewish Talmud, TD were construed as a prophecy for the impending death of a family member (Gutheil, 1951). The connection between TD and death was a common belief for many years, which Freud (1900) reacted to with irony, suggesting instead that TD represent sexual elements including masturbation and castration. Notably, however, Freud also referred to an assumption that had already existed in his time, according to which, TD were related to dental stimuli (in his view, this simple explanation was probably true, yet absolutely insufficient). Other interpretations of TD included Jung’s reported notion that TD in women represented childbirth (see Freud, 1900), as well as a more recent interpretation of TD as the fear of growing older (Schneck, 1967).

More recently, Yu (2012) found a correlation of TD with a dream content scale assessing somatosensory and motor experiences in dreams, such as falling, being chased, or flying (see Yu, 2010), and this correlation was stronger than that of TD with other dream content scales. Yu (2012, 2016) interpreted this scale as “sensorimotor excitement” and hypothesized, as Freud and his contemporaries did, that TD may be related to dental stimulation. Moreover, Revonsuo (2000) specifically hypothesized that TD are triggered by episodes of sleep bruxism, as the sensations elicited in the mouth are incorporated into the dream. This idea is in accordance with findings from modern sleep research on “incorporation”, specifically, studies on the effect of somatosensory stimuli on dream content using experimental manipulation (e.g., Dement and Wolpert, 1958; Nielsen, 1993). This line of research, showing that at times incorporation is possible, suggests that the origin of some dreams is most likely physiological rather than a direct or symbolic portrayal of psychological concerns. However, to the best of our knowledge, the hypothesis about the link between TD and dental irritation has never been empirically tested.

Dental irritation, stimulation, or tension during sleep may be considered as one of the indicators of teeth grinding or clenching according to the ICSD-2 diagnostic criteria for sleep-related bruxism (American Academy of Sleep Medicine, 2005). Dental irritation during the night is very common; it has been reported that 85–90% of the population grind their teeth at some point in their lives (ICSD; American Sleep Disorders Association, 1990). The prevalence of sleep bruxism tends to decrease with age and stands at 8.6% in the general population with no gender differences noted (Khoury et al., 2016). Nevertheless, it is worth noting that this may be an underestimation because people are often not aware of their habit to grit their teeth and/or lack a bed partner to draw their attention to the gnashing noise (e.g., Lavigne and Montplaisir, 1994; Kampe et al., 1997; Bader and Lavigne, 2000).

Kato and Lavigne (2010) listed clinical features for evaluation and diagnosis of sleep bruxism which include: self-report from sleep partners or parents who complain about grinding sounds, various conditions reported by bruxers upon awakening (e.g., jaw muscle discomfort, fatigue or stiffness, and tooth hypersensitivity), clinical observations (e.g., visual inspection), and miscellaneous (e.g., dental restoration failure or fracture). The possibility that many people grind their teeth, although they are not aware of it, may be the explanation for the commonness of TD, if they are in fact related to dental irritation. We hypothesize that TD may be related to such irritation, manifested in a sense of tenderness or tension in the teeth, gums or jaws upon awakening. The first goal of the present study was to examine in an exploratory manner whether there will be a significant correlation between TD and dental tension upon awakening. To explore the specificity of this relation (and its superiority over other correlations which may be influenced by self-report bias), we will also explore: (1) the correlation between TD and other types of sleep disturbances, and (2) the correlation between dental tension upon awakening and other typical dream themes.

In addition, in recent years it has been reported that an array of unusual dreams, including dreams of flying or falling, vivid dreams, recurring dreams, and dreams of dying, are components of a construct labeled “sleep experiences” (Watson, 2001). This construct is closely related to psychological symptoms and stress (Soffer-Dudek, 2017). TD have also been directly related to psychopathology (Coolidge and Bracken, 1984). This gives rise to an alternative, or supplementary, hypothesis, suggesting that TD may be related to psychological distress. This hypothesis may perhaps be viewed as the psychological/symbolical, rather than the physiological, interpretation of TD. Possibly, both hypotheses may be correct, especially since grinding teeth has been in itself perceived as a physical manifestation of stress and anxiety (Manfredini et al., 2005).

## Materials and Methods

### Participants and Procedure

This investigation was part of a larger study on dissociation and related constructs (Soffer-Dudek, 2018). In that study, data were collected from 303 undergraduate students in exchange for either course credit or reimbursement. Unfortunately, the two dental irritation items, central to this study (teeth grinding and teeth tension), were mistakenly omitted from the battery of questionnaires originally administered. Hence, they were administered 3 weeks later to those participants who agreed to complete them, which were *N* = 217 (72% of the sample). Independent samples *t*-tests demonstrated no significant differences between this subset of the sample and the rest of the participants on all study variables, including age, dream themes, psychopathology, sleep variables, and a chi-square test suggested that they were also not different in gender.

Missing data on study variables for the 217 participants were negligible (between 0 and 2.3% for any variable), and thus we did not implement any method for missing data completion; When missing data are lesser than 5%, any method for dealing with them would probably lead to the same results (Tabachnick and Fidell, 2007). However, we used the bootstrapping method (as detailed below), which automatically employs listwise deletion. Thus, our final sample for analyses consisted of *n* = 210 with full data (76.7% females, Mage = 23.4, *SD* = 1.43, and range: 18–28).

The participants signed up for a study labeled “Dissociation, attention, vulnerability, and resilience” via the institutional psychological experiments system. Through this system, they received a link to online survey software (Qualtrics, Provo, and UT) presenting the questionnaires. Ethical considerations of this study were approved beforehand by Ben-Gurion University’s institutional review board. After signing the electronic consent form, the participants completed the questionnaires in one of two possible fixed orders, which were counterbalanced between two randomly selected subsets of participants. Participants were instructed to respond to the questions as honestly as possible and to contact the researchers if they encounter any difficulties or concerns. The participants were subsequently debriefed regarding the purposes of the study. The procedure was completed in approximately 50 min.

### Measures

#### Dream Themes

The Dream Motif Scale (DMS; Yu, 2012) was designed to assess the lifetime frequency of experiencing particular dream content on a 5-point response scale (0 = never, 4 = once a month or more often). The full DMS consists of 100 dream themes, generating 14 subscales, each measuring a hypothesized “dream predisposition”. We used four items that were found by Yu (2012) to be highly correlated with the “Sensorimotor Excitement Scale”: item 12 (“falling”), item 18 (“your teeth falling out, losing your teeth, or your teeth rotting”), item 30 (“being unable to find, or embarrassed about using, a toilet”), and item 39 (“being smothered, and unable to breathe”). The DMS has excellent psychometric properties; the alpha reliability coefficients for all of its scales were reported to exceed the conventional level of 0.7. The structures of the DMS dimensions have been validated by item analyses, exploratory factor analyses, and confirmatory factor analyses.

#### Psychological Distress

The Brief symptom inventory (BSI; Derogatis and Melisaratos, 1983) is a 53-item scale assessing a wide range of self-reported psychological symptoms, including somatization, obsessive-compulsive symptoms, interpersonal sensitivity, anxiety, depression, hostility, phobic anxiety, paranoid ideation, and psychoticism, pertaining to the past month. It is useful for measuring general psychological distress. Participants estimated the frequency of their distress experiences in the past month on a 5-point scale (0 = not at all, and 4 = extremely). The authors document high test-retest and internal consistency reliabilities, and good evidence of convergent, discriminant and construct validity. A global BSI score was computed by averaging the 53 items, and subscale scores were computed by averaging the relevant items belonging to each scale. Cronbach’s alpha in the present study was 0.96 for the total score.

#### Dental Irritation

The two questions used in the present study to evaluate self-reported sleep bruxism were constructed based on: (1) the respondent’s awareness to grinding (item #1- teeth grinding: “I tend to grind my teeth while I am asleep”) and on: (2) vague sensations grinders may feel upon awakening, even if they are unaware of grinding, mentioned by the American Academy of Sleep Medicine (2005) as a diagnostic criterion of Sleep Bruxism (item #2- teeth tension: “Upon awakening I experience a sense of tenderness or tension in my teeth, gums or jaws”). Participants estimated the frequency of their dental irritation experiences pertaining to the past month, on a 6-point scale (0 = never or rarely; 1 = has happened a few times, but not in the last month; 2 = once in the last month; 3 = twice or three times in the last month; 4 = once in the last week; and 5 = more than once in the last week). The two items were significantly correlated [*r* = 0.63 (0.51, 0.72), *p* < 0.001], however, we used them separately in order to explore correlations when individuals are unaware of their grinding (represented by item #2). Single self-report items have been used to assess sleep bruxism in additional studies (Ohayon et al., 2001; Winocur et al., 2011; Shokry et al., 2016).

#### Sleep

Sleep quality was measured with the Pittsburgh Sleep Quality Index (PSQI: Buysse et al., 1989). The PSQI measures sleep quality of the past month. It consists of 19 items, summed up into seven components and a global score. Components include subjective sleep quality, sleep latency, sleep duration, habitual sleep efficiency, sleep disturbances, use of sleeping medication, and daytime dysfunction. Authors report acceptable measures of reliability and validity for this measure. We applied the Hebrew version of the PSQI which has been validated in Shochat et al. (2007). Cronbach’s alpha for the seven components in this study was 0.68. In this study, we used the global score (based on all 7 factors and representing poor sleep quality), as well as a single component which was of special interest to us: the sleep disturbances component. The latter was calculated as the mean score of ten sleep disturbances depicted in the questionnaire (e.g., cannot breathe comfortably, feel too hot, and had bad dreams). We labeled this scale general sleep disturbances, as opposed to dental disturbances, which are not included in the PSQI. We aimed to use this component in order to explore specificity of dental irritation as opposed to other types of disturbances to sleep. Notably, high scores on either of these variables (the global score or the general sleep disturbances component) indicate poor sleep quality.

### Data Analysis

Our aim in this study was to explore possible associations between variables, in a preliminary, cross-sectional design. All analyses were conducted with SPSS software (version 23). Because some of our variables (specifically, typical dream types and dental irritation items) were based on single items (with Likert-type response scales), we used Spearman rank-order correlation coefficients to explore relationships between the variables. We wish to note, however, that results were not significantly different when using Pearson zero-order correlation coefficients, or when using partial correlations controlling for gender^1^. Because the distributions of teeth grinding, teeth tension, TD, dreams of being smothered, toilet dreams, psychological distress and poor sleep quality were all positively skewed, we employed bootstrapping in order to test for significance without the need to assume normal distributions. The correlations were computed based on 1,000 bootstrapped resamples, and the 95% confidence intervals were computed using the bias-corrected and accelerated method.

## Results

**FIGURE 1:**
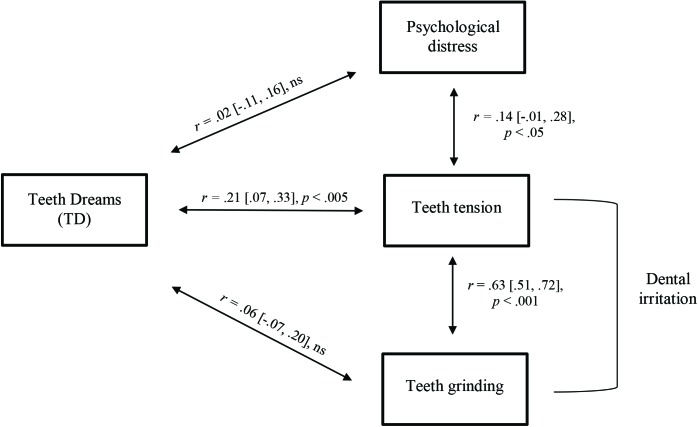
A figure depicting the main results: rank-order correlation coefficients linking teeth dreams (TD) with dental irritation (teeth tension and teeth grinding) and psychological distress.

**Table 1** presents means, standard deviations, and rank-order correlations for all study variables, specifically: the two dental irritation variables (i.e., teeth grinding and teeth tension), four typical dream themes (including TD, as well as falling, being smothered, and searching for a toilet), psychological distress, general sleep disturbances and poor sleep quality. As can be seen in the table, TD were positively associated with the teeth tension item, with a small-to-medium effect size [*r* = 0.21 (0.07, 0.33), *p* < 0.005], but were not associated with the item directly assessing teeth grinding [*r* = 0.06 (-0.07, 0.20), ns]. In fact, the latter item was not significantly associated with any of the other variables except for teeth tension (not even sleep quality or general sleep disturbances). Importantly, all three non-teeth typical dream themes were unrelated to dental irritation items, supporting the specificity of the TD-dental irritation relation. The hypothesis according to which psychological distress will be related to TD was not supported^2^ [*r* = 0.02 (-0.11, 0.16), ns], although psychological distress was related to teeth tension [*r* = 0.14 (-0.01, 0.28), *p* < 0.05]. Despite the statistical significance of the latter correlation, this association should be interpreted with caution, as the bootstrapped 95% confidence interval includes zero. Finally, TD were unrelated to sleep disturbances or sleep quality. The main results of the study are depicted in **Figure 1**.

**Table 1:** Means, standard deviations, and rank-order correlation coefficients of teeth dreams (TD), falling dreams, dreams of being smothered, toilet dreams, psychological distress, teeth grinding, teeth tension, poor sleep quality, and general sleep disturbances.

	1	2	3	4	5	6	7	8	9
(1) Teeth dreams (TD)	1.00								
(2) Falling dreams	0.34^∗∗^ [0.22, 0.43]	1.00							
(3) Dreams of being smothered	0.20^∗∗^ [0.05, 0.34]	0.27^∗∗^ [0.14, 0.38]	1.00						
(4) Toilet dreams	0.32^∗∗^ [0.18, 0.45]	0.22^∗∗^ [0.10, 0.33]	0.26^∗∗^ [0.11, 0.40]	1.00					
(5) Psychological distress	0.02 [-0.11, 0.16]	0.13^∗^ [0.02, 0.27]	0.27^∗∗^ [0.12, 0.39]	0.00 [-0.13, 0.14]	1.00				
(6) Teeth grinding	0.06 [-0.07, 0.20]	0.05 [-0.09, 0.18]	-0.01 [-0.15, 0.12]	0.02 [-0.11, 0.17]	0.02 [-0.13, 0.17]	1.00			
(7) Teeth tension	0.21^∗∗^ [0.07, 0.33]	0.08 [-0.06, 0.20]	0.11 [0.00, 0.24]	0.02 [-0.11, 0.16]	0.14^∗^ [-0.01, 0.28]	0.63^∗∗^ [0.51, 0.72]	1.00		
(8) Poor sleep quality	0.04 [-0.10, 0.17]	0.09 [-0.02, 0.22]	0.19^∗∗^ [0.07, 0.31]	0.05 [-0.08, 0.18]	0.50^∗∗^ [0.39, 0.61]	0.06 [-0.07, 0.20]	0.08 [-0.07, 0.23]	1.00	
(9) General sleep disturbances	0.09 [-0.05, 0.23]	0.13 [0.00, 0.26]	0.27^∗∗^ [0.13, 0.39]	0.14^∗^ [0.00, 0.26]	0.49^∗∗^ [0.38, 0.59]	-0.01 [-0.16, 0.13]	0.06 [-0.08, 0.21]	0.66^∗∗^ [0.59, 0.72]	1.00
*M*	0.54	1.65	0.36	0.47	0.71	0.84	1.04	0.68	5.36
*SD*	1.02	1.2	0.75	0.85	0.5	1.46	1.51	0.45	2.84

*^∗^*p* < 0.05, ^∗∗^*p* < 0.01. In brackets are the 95% bootstrapped confidence intervals, using 1,000 resamples, calculated with the bias-corrected and accelerated method and rounded down to two decimals*.

Other notable findings from these data were associations between dreams of being smothered (or unable to breathe) with psychological distress, general sleep disturbances, and poor sleep quality. Dreams of searching for a toilet were weakly associated with general sleep disturbances, and dreams of falling were weakly related to psychological distress.

## Discussion

The present study aimed to explore whether TD would be related to a physiological stimulus, specifically, dental irritation, or to a psychological or symbolic possible origin, specifically, psychological distress, or to both. Our first hypothesis, according to which TD would be significantly associated with dental irritation, was supported, with a small to moderate effect size. However, this relation reached statistical significance only for teeth tension, i.e., the item that indirectly assesses possible sleep bruxism of which the individual may be unaware, rather than the direct-assessment item pertaining to teeth-grinding. Although this may stem from the dearth of measures used in this study to assess bruxism, it may also represent support for the notion that most people may be unaware of their sleep bruxism yet may be aware of dental stimulation following the sensations in the organs located around and in the oral cavity after awakening (Rompré et al., 2007). We believe that the lack of effect with the direct-assessment item was brought about because many are unaware of grinding, and thus the variable probably had less variance in our sample, hindering the ability to identify relationships with it^3^. To the best of our knowledge, this is the first study to empirically demonstrate preliminary findings toward confirming the association between TD and dental irritation. It suggests one possible path through which the mind may distort somatosensory stimuli and incorporate them into dreams as a vivid and emotionally salient image (see Nielsen, 1993 for additional examples of such distortion)^4^.

In light of the small-to-medium magnitude of the relation, it is essential to examine the specificity of this association. Indeed, the results identified specificity in both directions. First, teeth tension was the only non-dream item correlated with TD, whereas general sleep disturbances, overall sleep quality, and psychopathology were unrelated to TD. Second, dental irritation was unrelated to other typical dream themes. To the extent that such specific results will replicate in future studies, this specificity supports the interpretation that TD follow dental irritation, or more broadly, that somatosensory stimulation influences and penetrates the content of dreams, albeit, taking on a vivid, emotional, and distorted, form (Nielsen, 1993). Importantly, however, causality may not be ascertained from our cross-sectional design. For example, it is also possible that TD bring about teeth clenching, which may cause tension upon awakening. Future research should employ a longitudinal diary design or induced awakenings in the sleep laboratory in order to establish chronological directionality.

Interestingly, the two dental stimulation items were unrelated to sleep quality or to general sleep disturbances. These results are consistent with Veldi et al. (2005) who found that sleep quality was unrelated to a number of sleep problems, including sleep bruxism. However, they do not appear to corroborate with Castroflorio et al. (2017) who showed a strong association between sleep bruxism and other sleep disturbances, especially snoring. Due to the preliminary nature of our study, further research is needed in order to gain a deeper understanding of the relation between bruxism and sleep quality, perhaps measuring these constructs as states rather than traits.

Somewhat surprisingly, in contrast to a previous finding (Coolidge and Bracken, 1984), at least in our preliminary study, TD were not associated with psychological distress at all, nor were there any correlations with specific psychological symptom subscales. This is in spite of the fact that psychological distress was indeed (weakly) related to sensitivity or tension in teeth, supporting the claim that anxiety and stress are related to teeth grinding (Ohayon et al., 2001; Manfredini et al., 2005; Kato and Lavigne, 2010). Again, to the extent that these preliminary findings will be replicated in future studies, this negates the potential confounding effect of psychological distress as an explanation for the relation of TD with teeth tension. However, it is important to consider that perhaps the BSI or its subscales are not the ideal measures to explore the hypothesized symbolic association between TD and psychological distress. For example, perhaps TD are related to some specific type of distress (e.g., fear of eating, and fear of speaking) which would be better assessed with specific symptom scales. This should be explored in future studies.

Importantly, we considered the possibility that our procedural technicality described in the Method section may have affected the magnitude of relations found; specifically, self-report measures administered concurrently may have inflated correlations due to context effects (Podsakoff et al., 2003). However, this means that context effects should have inflated the relation of TD with psychological distress (as they were administered concurrently), as compared to the relation of TD with dental irritation (as they were administered 3 weeks apart). Hence, the procedural mistake could have been an alternative explanation only to the extent that we would have found a stronger relation of TD with psychological distress than with dental irritation, but this was not the case; despite the possible context effect, we found that TD were related to dental irritation and were unrelated to psychopathology. Thus, our findings remain valid. Moreover, it is possible that a concurrent investigation would have resulted in a larger magnitude for the TD-dental irritation relationship.

Finally, another noteworthy finding which emerged in this study was an association of dreams of being smothered with both general sleep disturbances and with psychological distress. It seems that dreams of being smothered may have both somatic and psychological origins. On one hand, participants suffering from sleep-related respiratory pauses reported such dreams more often than controls (Yu and Thompson, 2016). On the other hand, MacFarlane and Wilson (2006) found that patients who report having breathing problems during waking dream of sensations of choking more often than patients with a sleep-related breathing disorder. This finding is in accordance with the continuity hypothesis and the idea that people are likely to dream about waking concerns and stressors (Schredl et al., 1998). Thus, it is possible that anxiety concerning suffocation may be one explanation for the correlation we found between psychological distress and dreams of being smothered. Furthermore, dreams of being smothered are reminiscent of a common phenomenon whereby individuals experience sleep paralysis while hallucinating that an evil presence (e.g., a demon or a spirit) sits on their chest and smothers them, with accompanying sensations of suffocation (Cheyne, 2003). Indeed, sleep paralysis is related to psychopathology (Sharpless and Barber, 2011). Moreover, sleep paralysis is an example of a sleep phenomenon which is influenced both by psychological factors (e.g., distress and social anxiety) as well as by physical sensations (e.g., shallow breathing, and body paralysis) (Solomonova, 2018). This phenomenon may suggest another pathway through which the sensation of suffocating during sleep may be connected to various types of psychopathology. Future work should focus on understanding and expanding the investigation of the origin of dreams of being smothered.

The current study has several limitations. First, our sample consisted of high-functioning college students and was biased toward women, which may restrict generalization to other populations. Second, in the current study we relied on single, self-report items to assess dental irritation. Although a considerable number of studies also used a method of single items to estimate bruxism (e.g., Ahlberg et al., 2003; Shokry et al., 2016), this may pose a threat to the validity and the reliability of the measure. Moreover, assessing sleep bruxism via self-report measures can yield inaccurate results; on the one hand, Bader and Lavigne (2000) stated that people are often not aware of having sleep bruxism and thus it may lead to an underestimation of the prevalence; yet, on the other hand, Maluly et al. (2013) found that self-reports may overestimate the diagnosis of sleep bruxism, despite the existing overlap between the objective and the subjective measures. Hence, future studies on the link between TD and dental irritation should examine sleep bruxism with a validated scale or with more objective measures, such as polysomnography (for example, see: Lavigne et al., 1996) and clinician observation. Third, the dearth of measures of both TD and physical complaints in this study limits our ability to identify the extent to which this relation is specific. In other words, while there was one correlation between TD and dental irritation, with a modest effect size, we did not assess other physical complaints that could further bolster or generalize this finding, or increase the percentage of explained variance; it is possible that TD are related to a number of aches and pains, and symbolize physical pain more generally as opposed to specifically being associated with dental irritation. Conversely, future studies may bolster the specificity of these findings by assessing additional types of teeth complaints, such as gum soreness, chronic toothaches, tooth bridges, gingivitis, or sensitive teeth. There may also be a possible relation between waking bruxism and TD. Fourth, as mentioned above, self-reported sleep bruxism was assessed approximately 3 weeks after the rest of the variables, which may be one of the reasons for the modest magnitude of correlation found between TD and dental irritation. The assessment after 3 weeks also resulted in a smaller sample for the study. Future studies should rely on larger and more diverse samples. Notably, however, this also may be viewed as a strength, as the main findings cannot be alternatively explained by context effects, because same-context variables (psychological distress, and sleep quality) were unrelated to TD, whereas a different-context variable (dental stimulation) was. Fifth, psychological distress, sleep quality, and dental irritation were assessed pertaining to the past month, while dream themes were assessed as a lifetime experience, in accordance with the original response scales of each of these questionnaires. Future studies should assess TD in a given time frame rather than assessing a life-time prevalence. Future studies will also benefit from assessing the constructs with a daily-diary design, rather than as retrospective traits. Notably, given the fact that psychological distress and dental irritation may both be transitory, it is likely that the use of different time frames (i.e., the assessment of dental irritation 3 weeks later) led to a reduction of the effect size between dental irritation and TD. Nevertheless, these limitations do not constitute an alternative explanation for our results, according to which, dental irritation was associated with TD whereas psychological distress was not. Another limitation of the present study is that we did not correct the alpha-level for multiple tests (although the Bonferroni correction is not suitable with interdependent measures, perhaps other methods could be applied). However, as we would expect 5% of comparisons to be statistically significant due to chance alone, this translates to less than one when looking at the 8 correlation coefficients calculated for TD. In addition, looking at the pattern of results, they do not seem to be random; TD was related to other types of unusual dreams and to teeth tension, in the expected directions. Thus, our results are probably not due to chance. Finally, future research may also explore the potential connections between several other sources of physical/somatic stimuli and other typical dreams, for instance flying, falling, or being frozen or unable to move (Yu, 2016). This may be an interesting way of studying the incorporation of sensory phenomena into dreams. Studies in the field of incorporation have usually focused on the prevalence of incorporation (i.e., the chances that a stimulus will be incorporated), using experimental methods; but much less is known on the dream mechanisms that distort the incorporated content. The present study preliminarily suggests that sensations of tension in one’s teeth during sleep may be translated by the sleeping consciousness to images of teeth rotting or falling out. Such distortion to an emotionally salient image may be related to the activity of the amygdala during REM sleep (Dang-Vu et al., 2007). Perhaps psychobiological research is needed in order to further understand why or how this translation comes about. Notably, to the extent that these results will replicate in future studies with at least moderate effect sizes, they may be of practical significance. Specifically, since it is common for people to be unaware of their habit to grind or clench their teeth during sleep, TD may perhaps serve as indicators of undiagnosed bruxism, and thus may be utilized by dentists as an aid for screening of dental problems.

To the best of our knowledge, the present study is the first to empirically validate the hypothesized connection between TD and nocturnal dental stimulation or irritation. The findings seem to imply that TD are most likely affected by physical sensations over the alternative hypothesis, according to which TD have a symbolic psychological meaning, representing psychological distress. Future studies are needed in order to replicate this effect (especially due to the small-to-medium effect size in this study) and expand this research line to other prevalent dream themes. Nevertheless, this preliminary study is a first step for understanding physiological effects on the production of common or universal dreams.

## Data Availability Statement

The raw data supporting the conclusions of this manuscript will be made available by the authors, without undue reservation, to any qualified researcher.

## Author Contributions

NR and NS-D designed and conceptualized the study, recruited the sample, collected and analyzed the data, conducted the literature search, and wrote the manuscript.

## Conflict of Interest Statement

The authors declare that the research was conducted in the absence of any commercial or financial relationships that could be construed as a potential conflict of interest.

## Footnotes

1 We hypothesized that gender may pose a confound, because women recall more dreams (Schredl and Reinhard, 2008), and report more psychological distress (Al-Issa, 1982), especially internalizing, rather than externalizing, mental health issues (Eaton et al., 2012). Thus, we attempted to control for gender in all analyses using partial correlations. Results remained unchanged.

2 Due to the non-significant correlation between TD and the BSI total score, we also explored BSI subscales to see if there were any specific symptom scales that would correlate with TD. However, none of the subscales correlated significantly with TD. Effect sizes ranged from *r =* -0.02 to *r =* 0.12.

3 Indeed, looking at the distribution of the teeth grinding variable, we noticed that it was very peaked, with high kurtosis (>2), whereas the teeth tension variable had normal kurtosis (<1), implying more variance in our sample.

4 Interestingly, prevalence rates presented in the Introduction section for TD (8.2%) and for Bruxism (8.6%) are very similar; possibly, this similarity stems from their connection. We thank the reviewer who brought this to our attention.
